# Exercise experiences in patients with metastatic lung cancer: A qualitative approach

**DOI:** 10.1371/journal.pone.0230188

**Published:** 2020-04-02

**Authors:** Pi-Hua Chang, Ching-Rong Lin, Yun-Hsiang Lee, Yi-Lin Liu, Gee-Chen Chang, Aasha I. Hoogland, Yeur-Hur Lai

**Affiliations:** 1 Department of Nursing, Taichung Veterans General Hospital, Taichung, Taiwan, Republic of China; 2 School of Nursing, College of Medicine, National Taiwan University, Taipei, Taiwan, Republic of China; 3 School of Nursing, College of Medicine, Chang Gung University, Taoyuan City, Taiwan, Republic of China; 4 Proton and Radiation Therapy Center, Department of Radiation Oncology, Chang Gung Memorial Hospital, Taoyuan City, Taiwan, Republic of China; 5 Division of Chest Medicine, and Comprehensive Cancer Center, Department of Internal Medicine, Taichung Veterans General Hospital, Taichung, Taiwan, Republic of China; 6 School of Medicine, Faculty of Medicine, National Yang-Ming University, Taipei, Taiwan, Republic of China; 7 Department of Health Outcomes and Behavior, Moffitt Cancer Center, Tampa, Florida, United States of America; 8 National Taiwan University Cancer Center, Taipei, Taiwan, Republic of China; Curtin University, AUSTRALIA

## Abstract

**Background:**

Patients with metastatic lung cancer can have severe cancer-related symptoms and treatment-induced side effects. Exercise is beneficial for patients with metastatic lung cancer; however, little information is available on guiding patients how to perform exercise during hospitalization. The purpose of this qualitative study was to understand exercise experiences in patients with metastatic lung cancer.

**Methods:**

Patients with metastatic lung cancer (n = 24) participated in face-to-face in-depth interviews at an inpatient ward of a medical center in central Taiwan. Interview transcripts were evaluated using narrative analysis to extract and validate themes.

**Results:**

Three primary themes were identified: (1) modifying exercise to maximize physical functions; (2) living with symptoms and frustration, but still exercising; and (3) doing exercise to sustain hopes, inner power, and life. Secondary findings included: (1) adopting walking as their main form of exercise because of its convenience; and (2) among patients with severe symptoms, adjusting exercise towards shorter time durations and shorter distances, slower speeds, and higher frequencies.

**Conclusions:**

The study found physically active lung cancer patients, although with metastatic condition, adjusted their exercise activities to balance disease and treatment-induced deteriorations and boost themselves to feel hope and fight for cancer. However, the results may not be applicable to physically inactive patients. Future research to explore experiences from those with even worse physical conditions and further helping them to take some mild exercise to enhance the positive side of cancer experiences are suggested.

## 1. Introduction

Lung cancer is one of the most commonly diagnosed cancers worldwide [1]. Over half of patients with lung cancer are initially diagnosed with metastatic lung cancer [2–4]. Patients with metastatic lung cancer often suffer from severe cancer-related symptoms and treatment-induced side effects, such as pain, fatigue, and dyspnea [5, 6]. These severe symptoms can impair cardiorespiratory function and physical capacity [7–9]. As a consequence, patients’ physical health can further deteriorate due to physical inactivity [10, 11]. Physical impairments can reduce health-related quality of life, which is of critical importance among patients with poor prognoses [12], such as patients with metastatic lung cancer. However, we noticed that some patients walked or swung arms slowly in the corridors even if they looked weakly during hospitalization.

Regular exercise in cancer patients has been recognized to improve quality of life [13], mitigate illness symptoms or treatment-induced side effects [14–16], improve functional capacity/cardiorespiratory fitness [17], and reduce mortality [18]. In previous studies, exercise programs, especially for patients with metastatic lung cancer, have been shown to improve physical fitness, functional capacity, symptom distress, emotional disturbance, and quality of life [11, 19–25]. Several systematic reviews on this topic have identified benefits of exercise for patients with advanced cancer, including metastatic lung cancer [9, 26–28]. It is unclear, however, how to optimally balance exercise considerations with cancer- and treatment-related side effects [29]. In Taiwan, patients with metastatic lung cancer seldom participate in professional exercise educations or classes. Instead, patients generally adhere to their past experiences and preferences as guide for modifying their exercise behaviors after cancer diagnosis [30, 31]. Therefore, we conducted a qualitative study to explore how Taiwanese patients integrate past exercise experiences into their exercise regimens.

The purpose of the present study was to better understand the views and experiences of exercise for hospitalized patients with metastatic lung cancer, both before and after diagnosis Knowing why patients persist in doing exercise, as well as how they adjust their exercise behaviors [32] under various physical or psychological conditions, is useful for making exercise recommendations appropriate for this vulnerable population.

## 2. Methods

### 2.1. Study design

We used a narrative-based qualitative approach with open-ended interviews to explore exercise experiences of patients. Narrative analysis is an effective way to understand less structured, yet rich and in-depth descriptions of thoughts, feelings, and meanings [33], and has been used in studies of cancer patients [34, 35] and their exercise behaviors [36]. In this study, narrative analysis facilitated exploration of the rationale underlying time-spent on exercise and on exercise activities [37, 38].

### 2.2. Study setting and participants

This study was conducted at an internal chest ward with 52 beds housed in a medical center with 1,500 beds in central Taiwan. This ward specializes in caring for adults with respiratory problems (lung and breathing) in which about 60% of them are diagnosed with lung cancer. Averages of two to three hospitalized patients/week with metastatic lung cancer receive diagnostic examinations, treatments, symptom management, or palliative care.

Purposeful sampling was used to identify potential participants [39]. The inclusion criteria were as follows: (1) older than age 20 years; (2) diagnosed with metastatic lung cancer; (3) spoke Chinese or Taiwanese; (4) admitted to the hospital; (5) either currently or past had irregular or regular exercise activities (i.e. having exercise habits) [40, 41] in the hospital or at home; and (6) understood the research purpose and agreed to participate in the study. The exclusion criteria were as follows: (1) diagnosed with stage I ~ IIIB lung cancer; (2) not admitted to the hospital; (3) reported no exercise before and after cancer diagnosis; (4) refused to participate in the study, (5) declined to audio-record the interview, and (6) physical infirmities (e.g., too sick to talk). All eligible patients were identified and referred to trained interviewer by two authors (PHC and GCC).

### 2.3. Ethics

Ethical approval was obtained from the Institutional Review Board of Taichung Veterans General Hospital, Taiwan (Certificate of approval: C10188). Patients provided written informed consent prior to participating in the study.

### 2.4. Procedures and data collection

Authors in this study were trained in qualitative research and interviewing at Master’s or Ph.D level. Each participant was interviewed face-to-face by a Master’s-level interviewer (YLL) who received training in qualitative research and interviewing. One author (YLL) approached eligible inpatients and explained to them the purpose and procedures of the study. After obtaining written informed consent, background information of each participant was recorded, and an interview was scheduled. Background information included the following participant characteristics (age, sex, marital status) and disease and treatment-related information, including time since diagnosis, metastasis sites, current cancer treatments, and the performance status using the Eastern Cooperative Oncology Group (ECOG) scale [42].

Methods of data collection were in compliance with the principles of narrative interviews [43, 44]. A quiet and comfortable interview room provided a peaceful and trustful ambiance for interviewing participants and caregivers. Participants agreed to share their experiences of exercising before and after their diagnosis of metastatic lung cancer. For those who agreed to participate in the study, the interview began with the following question: ‘can you talk about your thoughts and experiences on exercise?’ in accordance with an interview guide (Table 1). Each participant was interviewed individually for a duration of 30–45 minutes. All interviews were audio-recorded and transcribed within 48 hours. Transcriptions were all de-identified and stored electronically for analysis.

**Table 1:** Interview guide.

No.	Questions
**1.**	**Can you talk about your thoughts and experiences on exercise?**
	How did you feel about exercise before and after the diagnosis of lung cancer?
	Can you talk about the exercise you take in daily life and how do you do it?
	What are your thoughts or opinions about exercise?
**2.**	**How did you adjust your exercise behaviors in response to various physical or psychological conditions?**
	How did you adjust or modify your exercise behaviors in different situations?
	Can you talk about how you changed your exercise behaviors in your situations?
	Why did you continue or stop doing exercise?

### 2.5. Qualitative data analysis

We followed Floersch and associates’ principles of narrative analysis [45] and established the credibility of the analyses [43, 46]. Two authors (PHC and YLL) had working experience in the study setting for over 10 years and ensured the accuracy of the contents and contexts transcribed from the audio-recording after the interview and invited interviewees to confirm their transcribed messages [37, 47]. To gain details of the rich messages and thick descriptions from the interviews, we used analyst triangulation [45]: i.e., three authors (PHC, CRL, and YHL) were assigned to independently read, repeatedly reflect, constantly compare cancer- and exercise-related words, sentences and situations, and collaboratively discuss narratives to identify the findings consistent with participants’ experiences and semantic expressions. For disagreements, we checked and discussed with the interviewer (YLL) about the interview scenarios to represent participants’ thoughts or exercise experiences again. The themes and participants’ quotes were constructed [48] based on a drop-down list and data validation feature in the Excel software (Microsoft, 2016 version). Finally, the last author (YHL) confirmed and approved findings of the main themes, quotations, and interpretations.

Initially, 32 eligible participants with metastatic lung cancer were invited to participate in this study. Two participants refused to enroll; and two agreed, but their voices were too soft to be recorded. We interviewed 28 participants, four of which were excluded for having non-metastatic lung cancer (two in stage IB, one in stage IIIA, and one in stage IIIB of the disease) after rechecking the electronic medical records. Finally, data saturation was reached with the 24th patient with metastatic lung cancer with exercise experiences. This sample size of 24 was considered appropriate for constructing specific information about a specific phenomenon in a qualitative study [49]. The main themes were captured from the dialogues of all individual narratives and related contexts [43].

## 3. Results

Demographic and medical characteristics of the 24 participants (15 men, 9 women) with metastatic lung cancer are shown in Table 2. Their average age was 60.3 years (range: 49‒78). At the time of the interviews, participants had been diagnosed with metastatic lung cancer, on average, 17.3 months earlier. All but one participant was married (95.8%). Eleven participants had an ECOG score of 2 (45.8%). Grade 2 indicated that: (a) they were ambulatory and capable of all self-care but unable to carry out any work activities, and (b) they were up in > 50% of their waking hours. Most participants (70.8%, n = 17) had at least a single metastatic site, and 14 (58.3%) previously underwent chemotherapy.

**Table 2:** Characteristics of participants with metastatic lung cancer (*N* = 24).

Characteristics	mean(SD) or n(%)^a^
Age (years)	60.3 (9.1)
Time since diagnosis of metastatic lung cancer (months)	17.3 (15.1)
Gender	
Male	15 (62.5%)
Female	9 (37.5%)
Marital status	
Married or live with a partner	23 (95.8%)
Single	1 (4.2%)
ECOG^b^	
Grade 1	8 (33.3%)
Grade 2	11 (45.8%)
Grade 3	4 (16.7%)
Grade 4	1 (4.2%)
Metastatic site(s)	
One	17 (70.8%)
Two	6 (25.0%)
Three	1 (4.2%)
Cancer treatment modality	
Chemotherapy	14 (58.3%)
Radiotherapy	1 (4.2%)
Target therapy	1 (4.2%)
Palliative care (included symptom managements)	7 (29.2%)
Diagnosed but not yet treated	1 (4.2%)

^a^ mean(SD) or n(%): mean(SD) indicated continuous values and n(%) indicated categorical values.

^b^ECOG: the Eastern Cooperative Oncology Group.

## 4. Narrative findings

### 4.1. Thematic findings

To summarize exercise behaviors, we identified three primary themes: (1) modifying exercise such as shortening time, lowering speed, narrowing field, or increasing frequency to maximise physical functions; (2) living with frustrations and symptoms such as breathlessness, fatigue, pain, or metastasis-induced immobility, but still exercising; and (3) doing exercise to sustain hopes, inner power, and life, also encouraged by self-confidence, social supports, and religious belief.

#### Theme 1: Modifying exercise to maximize physical function

Participants felt or thought that exercise capacity and related symptoms were like the warning signals of cancer and treatments. For participants with metastatic lung cancer, maintaining regular exercise was not easy due to the unpredictable progression of the disease and hard-to-tolerate treatments (patients 2, 6, 24). Some participants were unable to walk too long or too far for fearing that once they left their home, they would not be able to return (patients 2, 13). Several participants modified exercise frequency, intensity, time/duration (patients 2, 3), and types (patients 3, 4, 13) to maximize physical functioning. Other participants thought continuing to exercise would help them feel energetic and healthy again (patients 13, 24), especially in places of good air quality, like in the forest, rural areas (patients 4, 6), or a park. One patient indicated that their preferred strategy involved changing the timing and location of exercise as a function of the disease or treatment-induced symptoms they were experiencing (patient 2).

*“I was afraid of getting infected from others after chemotherapy. So I went out walking early in the morning. I did not feel breathless while exercising, because I just walked around my neighborhood park, which was not far from my house, and I walked for only about 10 minutes*.*”* (Patient 2, man)

*“When I had enough energy, I did not get tired easily, and I could get up earlier to do Wai Tan Kung (a form of Chinese exercise) with friends. However, if my condition were not good, I would not complete the whole session, but I still did a part of the exercise*.*”* (Patient 3, woman)

*“After I got cancer, I moved to a rural area because it was suitable for walking in the early morning. When the sun had not risen yet, the concentration of phytoncide and negative ions were high in the forest. I could breathe fresh air and increase the vital capacity of my lungs*.*”* (Patient 4, man)

*“Because of my disease and treatments, I sometimes felt breathless. However, I still climbed mountains, took more breaks to complete the whole exercise*! *The fresh air could be helpful for my breathing*.*”* (Patient 6, woman)

*“I hoped to walk longer and farther, but I also worried about a lack of energy and not being able to walk back. So, I walked with my bicycle and alternatingly walked and biked. Therefore, if I was too tired to walk home, I rode home. This alternative method kept me walking farther and exercising longer*.*”* (Patient 13, woman)

*“I want to try and see if I can increase my lung capacity. I want to know if increasing the physical capacity and stamina can make my body healthier*.*”* (Patient 24, man)

#### Theme 2: Living with symptoms and frustration, but still exercising

Although many participants with metastatic lung cancer tried to keep exercising after diagnosis, cancer or treatment-induced symptoms and adverse effects negatively impacted their ability to exercise (patients 7, 8, 9). As a result of emergent physical symptoms/conditions, participants noted that they had to change the distance, speed, duration, and nature of their exercise (patients 5, 7, 8, 10). For one patient (patient 21), an inserted central line was one reason she changed her behaviors. Some participants felt that their exercise capacity fluctuated and gradually declined as their disease and treatments progressed (patients 14, 16, 21), and the places in which they exercised changed from open spaces to indoors (patient 15). Two participants also worried about getting sick in unstable weathers, like when it was especially cold, windy, rainy, or hot (patients 2, 20).

*“If it was really cold and windy, I did not have the courage to go out (exercise). But winter was coming soon*!*”* (Patient 2, man)

*“I feel breathless*! *So I want to walk. My body was full of toxins of chemotherapy. When I walked, I sweated. The more I walked, the less breathless I felt. Because the toxins were excreted from my body, I felt that my body is much healthier*.*”* (Patient 5, man)

*“During the treatment period, I felt very distressed, weak, and exhausted. Only when I walked outside and looked at the remotely beautiful scenery, I felt better and relaxed*.*”* (Patient 7, man)

*“After chemotherapy, I felt fatigued more moderate than before. I needed to rest for about 2–3 hours after exercise to gain physical strength back*!*”* (Patient 8, man)

*“Of course, I felt very disappointed when my condition was deteriorated fast. I felt breathless and walked intermittently even from bedside to the toilet*.*”* (Patient 9, man)

*“When I felt breathless because of pleural effusion, I did not need any rest; I just walked a little slowly*.*”* (Patient 10, man)

*“After having the problem [lung cancer], I could not walk fast*! *I felt that I did not have as much physical strength as before [pre-illness]. When I did not feel well or could not walk too fast, I just walked at my own pace. However, I wished I could walk quickly again*.*”* (Patient 14, woman)

*“I needed a cane to go out walking because of spine metastasis and leg weakness, so I still thought, “could I go out*? *I felt very stressful when the stranger looks at me. It was inconvenient to meet friends or to do something else. I could only be at home and go walking around in my local neighborhood*.*”* (Patient 15, man)

*“Cancer cells had taken a bite out of my bone (patient pointed at her lumbar spine). So, I could only walk approximately 600 meters at most*.*”* (Patient 16, woman)

*“I liked playing golf, but the Port-A (an artificial vascular device) made me very painful. I could not do rotational motions on my shoulders*! *Sometimes it [exercise] made me feel tired. I thought it might increase my immune function. So, I needed to take several breaks during my walking. I still kept walking*.*”* (Patient 21, woman)

*“If I felt breathless or if it was too cold outdoors, I am not able to go out for walking*.*”* (Patient 20, man)

#### Theme 3: Doing exercise to sustain hopes, inner power, and life

For some patients, exercise aroused cancer patients’ hopes for surviving. For example, one participant expected to improve their physical status and have better chances for survival (patient 8). For other participants, doing exercise to maintain good physical energy helped patients to feel they could overcome cancer (patients 2, 6). One participant noted that they changed their exercise behaviors and to maintain physical strength and build up self-confidence (patient 16). Sources of social support (e.g., family members, spouses, friends), and physicians were all critical sources support that encouraged patients to exercise (patients 2, 9, 16). One participant emphasized that religious beliefs gave her the willpower to engage in exercise (patient 21).

*“Exercise was good for my physical health. I went walking with my wife, or we took a walk together, and it made me and my family happy and peaceful*!*”* (Patient 2, man)

*“I thought it (lung cancer) has helped me realize how I want to live. I thought that if I did not get this disease, I would go to work early in the morning and work too late to exercise. Now, I arranged more time for climbing mountains and did what I wanted to do. I could choose the most difficult trails in the mountains to test my physical strength and estimate how much my body had improved and my cancer status*.*”* (Patient 6, woman)

*“You could not survive without exercise*! *When you exercised, you could get bad things out of your body*.*”* (Patient 8, man)

*“I accepted others’ suggestions*: *You had to change your lifestyle and increase your lung capacity after getting cancer. My coworkers were very kind to me. If I walked tired, they told me to rest on the sides*.*”* (Patient 9, man)

*“After surgery, my doctor advised me to exercise (walk)*! *When I felt well after exercise, I felt more confident in challenging cancer*.*”* (Patient 16, woman)

*“I had a strong will to live longer because I knew life is precious. In my exercise group, they all encouraged me and told me that I was very courageous. The power was from my Bodhisattva [Pusa, a Chinese god]. If I could live more than five or six years, I hoped to keep walking and became a pious Buddhist. Exercise made me feel powerful, as well. At least, it was what I believed*.*”* (Patient 21, woman)

### 4.2. Secondary findings

**Fig 1:**
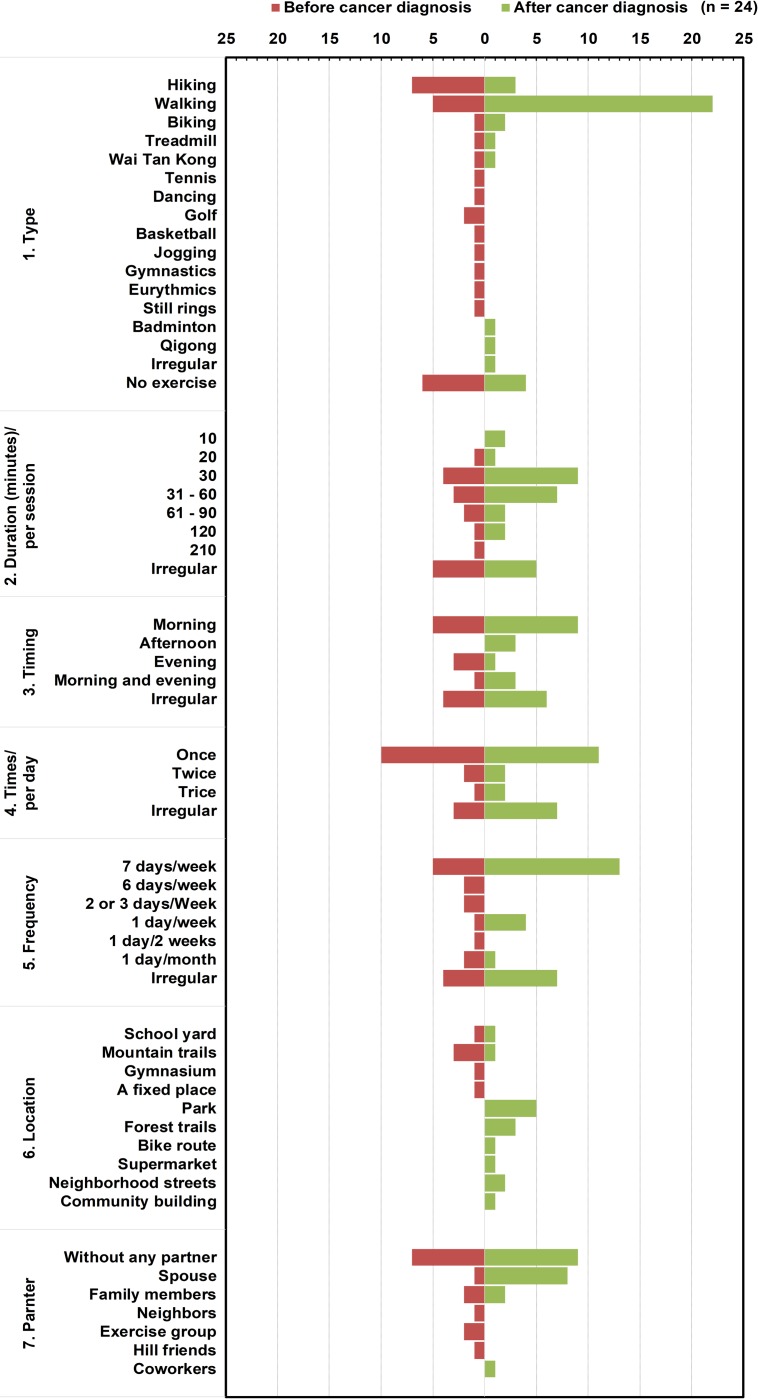
The components of exercise extracted from participants with metastatic lung cancer. Irregular is defined that participants adopted various duration, timing, times, and frequencies in combination with exercise types, locations, or accompanying partners.

From interview transcriptions, we classified various components of exercise as shown in Fig 1. Secondary findings included: (1) adopting walking as their main form of exercise because of its convenience; and (2) among patients with severe symptoms, adjusting exercise towards shorter time durations and shorter distances, slower speeds, and higher frequencies.

Walking was the most common form of exercise, both before and after the cancer diagnosis. Nine participants exercised at levels of moderate-to-heavy exertion and resistance before cancer diagnosis, but after cancer diagnosis, they stopped exercising altogether. Following cancer diagnosis, most participants adapted exercises according to their physical conditions, such as mild-to-moderate exertion with individualized durations, frequencies, modes, timings, and locations, such as home-based rehabilitation programs. Seven participants who had had no previous exercise habits started walking after diagnosis. When feeling well, most participants wanted to do exercise without a partner, but some liked exercising with a spouse or family members. A subset of participants often adopted ‘irregular’ durations, timings, times, and frequencies in combination with exercise types, locations, or accompanying partners with the hope to maintain a healthier lifestyle. Finally, four participants stopped exercising after diagnosis because of their declining physical or bedridden conditions.

## 5. Discussion

The principal purpose of our present study was to understand patients’ exercise experiences. Findings were based on qualitative analyses of interviews with patients with metastatic lung cancer, which examined the relationship between cancer and exercise. Our study is the first, to our knowledge, to explore exercise experiences in non-English speaking patients. Our narrative data would be helpful to develop patient-centered exercise instructions and encourage patients with no exercise to start or to avoid stopping exercise.

### 5.1. Methodological considerations

Results from this narrative analysis expand on existing literature examining patients with advanced lung cancer [30, 50–52]. This study focused on daily physical activities, which were modified by participants themselves according to their physical, psychological, social, and spiritual capabilities, and revealed that patients reported diverse ranges of exercise. In addition, previous studies mostly examined the effects of designed exercise programs for participants with metastatic lung cancer [9, 19–25, 51, 53]. In contrast, our narrative study put emphasis on exploring situational exercise experiences before and after a cancer diagnosis.

Through a simple interview guide and a reliable setting to gather rich information from participants’ narratives, we reached the conclusion that participants with metastatic lung cancer have strong exercise intentions to adjust their exercise behaviors along with their treatment plans [54], in spite of cancer- and treatment-related symptoms and side effects. The findings of this narrative analysis strongly suggest that a standardized exercise guideline of a total 150-minute moderate-intensity exercise on a weekly basis might not be readily achievable for patients with late-stage or metastatic lung cancer [11, 37, 55]. In fact, exercise guidelines, such as resistance, balance, and stretching exercise programs, were suggested to be customized for fitting cancer patients with metastatic disease or severely weakened physical conditions [12, 23, 56–60].

### 5.2. Primary findings

Exercise helped our participants with metastatic lung cancer surmount adversities due to their declining physical capacity in a brave manner [61] and continue their treatment plans [12]. In our study, participants believed that their expectations of exercise were not only achievable [52], but also boosted themselves to be healthy cancer survivors. For those who had no previous exercise behaviors, they also accepted suggestions from other people and began exercising either after diagnosis or after starting treatment [50]. As caused by physical capacity that declined relative to their past conditions, one should recognize that symptoms such as breathlessness, weakness, and fatigue, were usually taken as early warning signals of the disease [29].

In addition, our participants experienced unique sensations of breathing fresh air and increasing vital lung capacity during exercise, even though they did not show a direct effect on the lung function. Since no evidence to support irregular exercise can enhance lung capacity, health care providers should support patients’ positive feelings as well as educate them the correct concepts about exercise. Similar situation was also found in one of the participants who reported the thought that exercise could excrete toxins of chemotherapy through sweating. In the experiences from both cases suggest that some patients may have high or even incorrect expectations about the benefits of exercise. Health care professionals should support patients, but, in the meantime, bring them more correct information about taking exercise. Furthermore, exercise should be individually prescribed and tailored based on symptoms, goals, and preferences. It would be necessary to instruct participants about the strategies to adjust exercise intensity, duration, type, or environment, to fit their physical conditions, and to prevent them from over-training [14].

Most of our participants indicated that they always wished they could walk for longer distances and for longer durations, although they understood cancer could worsen their body conditions drastically. According to our findings, exercise capacity was one of the crucial signals of maintaining their hopes of survival [62, 63]. With reductions of the intensity or duration of exercise, exercise became possible when participants felt stronger to fight cancer and treatment-related symptoms. We noticed that such enhanced hopes were found, even in those diagnosed with metastatic lung cancer [62]. Those participants who exercised after being diagnosed with cancer or receiving anticancer treatments also believed their inner power might have driven them to become healthier. One of our female participants said that religious belief was important in maintaining her exercise, a finding that is consistent with other studies on healthy people [64] and other cancer patients [65]. Consistent with previous studies [9, 32], we found that social supports from family, friends, and physicians were also needed in helping patients to change their exercise behaviors, especially for older patients with advanced cancer.

### 5.3. Secondary findings

We found that walking was the most acceptable form of exercise for our participants, because it required low exertion, no requirement, was self-paced, low-cost, and not limited in time and space [14, 31]. Our participants sometimes treated their walking performance as an indicator of their symptoms, level of physical mobility and cancer progression. This finding is consistent with previous studies using 6-minute walking distance as an objective outcome in exercise testing and interventions [55, 66–69]. In clinical practice, for physically weaker populations, suitable amount of exercise is recommended to prevent over-training and over-exertion [14, 66, 70].

Exercise is an effective way to balance internal homeostasis and body coordination [29]. In our study, we found seven different components of exercise reflected the diversity of contents and situations in exercise, namely: type, duration, timing, times, frequency, location, and partner. An interesting phenomenon was that participants often changed duration, timing, times, and frequency according to their self-assessed effects rather automatically. The physiological responses and sensations would protect the subjects from the deleterious effects of exercise and make the subjects adjust their exercise strategies [29]. For example, the type of exercise varied from light to heavy in intensity, encompassing aerobic activity, resistance exercise, balancing, and stretching. Therefore, we classified this type as ‘irregular’ because of quantitative and qualitative factors that could compromise exercise capabilities of patients with metastatic lung cancer [9, 14].

In summary, for these active patients with metastatic lung cancer adjusted their exercise activities to strengthen their psycho-spiritual power while faced adversities of cancer and treatments. Furthermore, patients may even benefit more from their family members or caregivers receiving education on how to prepare patients to fit a balanced exercise schedule, by adjusting its type, intensity, speed, and duration [15, 28, 71].

### 5.4. Limitations of the study

Several limitations of our study are described below. First, our sample included patients who took exercise activities either before or after a diagnosis of metastatic lung cancer. The findings may not be generalizable to patients who have not engaged in physical activity or who are in early stage lung cancer patients. Second, it is unclear to what extent social desirability influenced the narrative data obtained from patients reporting retrospectively on their exercise experiences before cancer diagnosis. Third, limited to the cross-sectional narrative study design, we were unable to explore the trajectory of exercise experiences across cancer process due to not flowing of the situational changes of disease, symptoms, exercises adjustment and patients’ feelings across time. Thus, a longitudinal exploration of patients exercise experiences in lung cancer patients would be needed in the future. Finally, in this study, we only explored patients who either had exercise before or after cancer diagnosis, we did not explore those who never do exercise and who are in very worse conditions without physically active. Future study would be also needed to expand the scope to them and to help them develop acceptable exercise activities and enhance the positive side of cancer process.

## 6. Conclusions

In this study, physically active metastatic lung cancer patients adjust their exercise behaviors to balance disease and treatment-induced deteriorations and boost themselves to fight cancer and remain hope. Health care professionals should further expend the results to better help patients with metastatic lung cancer to develop suitable exercise, not limited to walking, to enhance the positive side of cancer process. Future studies should also expand to those physically inactive lung cancer patients to explore their experiences and further support them.

## Supporting information

S1 Table Interview guide. (DOCX)

S1 Data (DOCX)

S2 Data (DOCX)
