# Supplementary material for: Exercise experiences in patients with metastatic lung cancer: A qualitative approach
Source: PLoS One. 2020 Apr 2;15(4):e0230188. doi: 10.1371/journal.pone.0230188 (PMC7117721; doi:10.1371/journal.pone.0230188)
Supplement: S1 Table — (DOCX) [file pone.0230188.s001.docx]

**The Chinese version**

**表1** 轉移性肺癌病人運動經驗訪談指引

| 編號 | 問題 |
| --- | --- |
| **1.** | **您可以談一談對運動的想法和經驗嗎?** |
|  | 您對運動的想法和看法是什麼? |
|  | 您可以談一談您的運動在每天生活中的狀況? |
|  | 您可以描述一下做這些運動的情況嗎?以及如何進行? |
| **2.** | **針對在不同的身體或心理狀況時，如何調整您的運動行為?** |
|  | 您覺得得到肺癌前、後，對運動的感覺如何?為什麼改變? |
|  | 在不同情況下，您如何調整或修正您的運動行為? |
|  | 您可以談一談在您當時的狀況，您如何改變您的運動行為? |
|  | 為什麼您會繼續或停止運動? |

**The English version**

# Table 1. Interview guide

| **No.** | **Questions** |
| --- | --- |
| **1.** | **Can you talk about your thoughts and experiences on exercise?** |
|  | How did you feel about exercise before and after the diagnosis of lung cancer? |
|  | Can you talk about the exercise you take in daily life and how do you do it? |
|  | What are your thoughts or opinions about exercise? |
| **2.** | **How did you adjust your exercise behaviors in response to various physical or psychological conditions?** |
|  | How did you adjust or modify your exercise behaviors in different situations? |
|  | Can you talk about how you changed your exercise behaviors in your situations? |
|  | Why did you continue or stop doing exercise? |
