# Supplementary material for: Exercise experiences in patients with metastatic lung cancer: A qualitative approach
Source: PLoS One. 2020 Apr 2;15(4):e0230188. doi: 10.1371/journal.pone.0230188 (PMC7117721; doi:10.1371/journal.pone.0230188)
Supplement: S1 Data — (DOCX) [file pone.0230188.s002.docx]

Manuscript Number:  [PONE-D-19-19518] R2

**Exercise experiences in patients with metastatic lung cancer: A qualitative approach**

Consolidated criteria for reporting qualitative studies (COREQ): 32-item checklist

| **No** | **Item** | **Guide questions/description** | **Page/line(s)** |
| --- | --- | --- | --- |
| **Domain 1: Research team and reflexivity** | | |  |
| Personal Characteristics | | |  |
| 1. | Interviewer/facilitator | Which author/s conducted the interview or focus group? | The fourth author (YLL) conducted the interview.  Page 7/lines 136-137 |
| 2. | Credentials | What were the researcher's credentials? *E.g. Ph.D., MD* | PhC: 1  Ph.D.: 3  MD. Ph.D.: 1  Master: 1  Title page |
| 3. | Occupation | What was their occupation at the time of the study? | Title page |
| 4. | Gender | Was the researcher male or female? | The researchers were five females and one male. |
| 5. | Experience and training | What experience or training did the researcher have? | Five researchers had received qualitative research training in the Master and Ph.D. program in Nursing.  Page 7/lines 135-136 |
| Relationship with participants | | |  |
| 6. | Relationship established | Was a relationship established prior to study commencement? | Yes.  Two authors (PHC and YLL) have still worked in the study ward.  Page 8/lines 156-159 |
| 7. | Participant knowledge of the interviewer | What did the participants know about the researcher? e*.g. personal goals, reasons for doing the research* | Yes.  Two authors (PHC and YLL) have still worked in the study ward, and participants knew them.  Page 8/lines 156-159 |
| 8. | Interviewer characteristics | What characteristics were reported about the interviewer/facilitator? e.g. *Bias, assumptions, reasons and interests in the research topic* | The interviewer is one of the authors and perceived interested in the research topic and works at the study ward.  Page 7/lines 135-136  Page 8/lines 156-159 |
| **Domain 2: study design** | | |  |
| Theoretical framework | | |  |
| 9. | Methodological orientation and Theory | What methodological  orientation was stated to underpin the study? *e.g. grounded theory, discourse analysis, ethnography, phenomenology, content analysis* | Narrative analysis.  Page 6/lines 106-111 |
| Participant selection | | |  |
| 10. | Sampling | How were participants selected? *e.g. purposive, convenience, consecutive, snowball* | Purposeful sampling.  Page 6/lines 119 |
|  |  |  |  |
| 11. | Method of approach | How were participants approached? e*.g. face-to-face, telephone, mail, email* | We used a face-to-face interview to approach every participant.  Page 7/lines 136 |
| 12. | Sample size | How many participants were in the study? | Initially, 32 eligible participants were invited to participant in the study. Finally, a total of24 participants were in the study.  Page 9/lines 169-174 |
| 13. | Non-participation | How many people refused to participate or dropped out? Reasons? | Two participants refused and six participants were dropped out.  Page 9/lines 171-173 |
| Setting | | |  |
| 14. | Setting of data collection | Where was the data collected? e*.g. home, clinic, workplace* | The setting was in the hospital.  Page 6/lines 114-116  Page 8/lines 145-146 |
| 15. | Presence of non-participants | Was anyone else present besides the participants and researchers? | Yes.  It was depended on participants and relative members because our study did not involve personal privacy issues.  Page 8/lines 146 |
| 16. | Description of sample | What are the important characteristics of the sample? *e.g. demographic data, date* | Page 9/lines 181-188 &  Table 2 |
| Data collection | | |  |
| 17. | Interview guide | Were questions, prompts, guides provided by the authors? Was it pilot tested? | Yes.  Page 8/line 148-149 & Table 1  No, it was not a pilot test. |
| 18. | Repeat interviews | Were repeat interviews carried out? If yes, how many? | No.  We did not repeat interviews. |
| 19. | Audio/visual recording | Did the research use audio or visual recording to collect the data? | Yes.  Page 8/line 151, 158 |
| 20. | Field notes | Were field notes made during and/or after the interview or focus group? | Yes.  Some field notes were square brackets as shown in Table 3. For example, The Wai Tan Kung is a form of Chinese martial art exercise (patient 3, woman). |
| 21. | Duration | What was the duration of the interviews or focus group? | Yes.  Page 8/lines 150 |
| 22. | Data saturation | Was data saturation discussed? | Yes.  Page 9/line 173-174 |
| 23. | Transcripts returned | Were transcripts returned to participants for comment and/or correction? | Yes.  Page 8-9/lines 158-159 |
| **Domain 3: analysis and findings** | | |  |
| Data analysis | | |  |
| 24. | Number of data coders | How many data coders coded the data? | Two data coders.  Page 8/lines 156-159 |
| 25. | Description of the coding tree | Did authors provide a description of the coding tree? | Yes.  Page 8/lines 162-164 |
| 26. | Derivation of themes | Were themes identified in advance or derived from the data? | Yes.  Page 9/line 159-165 |
| 27. | Software | What software, if applicable, was used to manage the data? | Yes.  The Excel Software (2016 version).  Page 8/lines 166-167 |
| 28. | Participant checking | Did participants provide feedback on the findings? | Yes.  Page 8/lines 158-159 |
| Reporting | | | |
| 29. | Quotations presented | Were participant quotations presented to illustrate the themes / findings? Was each quotation identified? e*.g. participant number* | Yes.  Table 3 |
| 30. | Data and findings consistent | Was there consistency between the data presented and the findings? | Yes.  Pages 9-16/lines 193-348 |
| 31. | Clarity of major themes | Were major themes clearly presented in the findings? | Yes.  Pages 9-15/lines 193-329 |
| 32. | Clarity of minor themes | Is there a description of diverse cases or discussion of minor themes? | Yes.  Pages 15-16/lines 331-348 |
