# Supplementary material for: Exercise experiences in patients with metastatic lung cancer: A qualitative approach
Source: PLoS One. 2020 Apr 2;15(4):e0230188. doi: 10.1371/journal.pone.0230188 (PMC7117721; doi:10.1371/journal.pone.0230188)
Supplement: S2 Data — (DOCX) [file pone.0230188.s003.docx]

**Exercise experiences in patients with metastatic lung cancer: A qualitative approach**

- Three primary themes were identified: (a) modifying exercise to maximize physical functions; (b) living with symptoms and frustration, but still exercising; and (c) doing exercise to sustain hopes, inner power, and life.
- Secondary findings included: (a) adopting walking as their main form of exercise because of its convenience; and (b) among patients with severe symptoms, adjusting exercise towards shorter time durations and shorter distances, slower speeds, and higher frequencies.
- Patients with metastatic lung cancer adjust their exercise activities to balance disease and treatment-induced deteriorations and boost themselves to feel hope and fight for cancer.
